# Acetylcholinesterase: old questions and new developments

**DOI:** 10.3389/fnmol.2012.00101

**Published:** 2013-01-09

**Authors:** Karl Tsim, Hermona Soreq

**Affiliations:** ^1^Life Science and Center for Chinese Medicine, The Hong Kong University of Science and TechnologyHong Kong, China; ^2^The Edmond and Lily Safra Center for Brain Sciences, The Hebrew University of JerusalemJerusalem, Israel

The family of cholinesterases, including acetylcholinesterase (AChE) and butyrylcholinesterase (BChE), has been of significant interest to neuroscientists for several decades. Cholinesterases play an essential role in mediating neurotransmission in cholinergic synapses, which control the higher brain functions such as learning and memory; they also are causally involved in controlling nerve-muscle communication and muscle activities, and serve as an important component of the physiological differences characteristic of certain pathological conditions. A notable example is Alzheimer's disease, where premature death of cholinergic neurons has been the basis for drug development so that the leading therapeutic agents available to date are targeted to inhibit cholinesterase activities. AChE and BChE were initially identified as efficient hydrolases that possess the capacity to rapidly hydrolyze acetylcholine and butyrylcholine, respectively; and AChE presents more rapid and far more selective potency for such hydrolysis compared to BChE. Throughout the last 40 years, multiple studies of cholinesterases led to an exponential increase in the effort to explore their functions, structures and physiological roles in health and disease. The resultant findings strongly suggest vital roles for cholinesterases both in the nervous system and in other body systems, with non-neuronal functions such as the regulation of apoptosis and inflammation being a relatively new focus of interest. This vast interest, coupled with the rapid increase in the availability and power of different research tools allow far deeper understanding of the structure and functions of AChE and BChE in different disciplinary terms, including cellular and molecular biology, genetics and epigenetics, physiology, structure-function relationships at an atomic resolution level, the chemical mechanisms of enzymatic inhibition, pharmacology, drug development, disease therapy, etc. However, in spite of this massive multi-disciplinary effort, numerous critical and core questions of AChE and BChE are yet not fully answered, and some have never been addressed as yet. As two of the many enthusiastic scientists in this field, we believe that the current surge in cholinesterase research will further expand in the near future. Therefore, it has been an honor to co-edit this E-book, entitled “Acetylcholinesterase: old questions and new developments.” This volume includes pertinent research articles by 11 leading groups, worldwide (Carvajal and Inestrosa, 2011; Chen et al., 2011; García-Ayllón et al., 2011; Hanin and Soreq, 2011; Bronicki and Jasmin, 2012; Durrant et al., 2012; Falugi and Aluigi, 2012; Gilboa-Geffen et al., 2012; Gnatek et al., 2012; Shehadeh Masha'our et al., 2012; Zhang and Greenberg, 2012). It covers the translational and post-translational modifications of cholinesterases, their developmental expression patterns in different species, their links to inflammation-related pathologies and their role in apoptotic processes. At the molecular level, one can find in this volume a reference to the microRNA regulation of cholinesterases as well as to the trans-acting factors governing the metabolism of cholinesterase mRNA. Also, the links of cholinesterases with inherited neuromuscular diseases as well as with Alzheimer's neuropathology and therapeutics is covered from both the physiological and structural points of view. Thus, this volume provides a glimpse into the most up-to-date research information in the diverse field of cholinesterases, as well as an important insight for outlying future research directions in this topic.
